# A qualitative study on the intersectional social determinants for indigenous people who become infected with HIV in their youth

**DOI:** 10.1186/s12939-017-0625-8

**Published:** 2017-07-21

**Authors:** Roberta L. Woodgate, Melanie Zurba, Pauline Tennent, Carla Cochrane, Mike Payne, Javier Mignone

**Affiliations:** 1 College of Nursing, Rady Faculty of Health Sciences, University of Manitoba, 89 Curry Place, Winnipeg, MB R3T 2N2 Canada; 2 Assembly of Manitoba Chiefs, 200-275 Portage Ave, Winnipeg, MB R3B 2B3 Canada; 3 Nine Circles Community Health Centre, 705 Broadway Avenue, Winnipeg, MB R3G 0X2 Canada; 4 Dept. of Community Health Sciences, Max Rady College of Medicine, Rady Faculty of Health Sciences, University of Manitoba, 307 Human Ecology Building, 35 Chancellors Circle, Winnipeg, MB R3T 2N2 Canada

**Keywords:** HIV/aids, Indigenous, Intersectionality, Social determinants, Youth

## Abstract

**Background:**

Indigenous young people are currently highly overrepresented in the HIV epidemic in Canada, especially in the Prairie Provinces, such as Manitoba. Understanding HIV-vulnerability in Indigenous peoples must begin with understanding that social determinants are intersectional and linked to the historical legacy of European colonization. In this paper findings that detail the influence of the intersectional social determinants on Indigenous people who become infected with HIV in their youth are presented.

**Methods:**

The qualitative research design of phenomenology was used as it afforded the opportunity to understand Indigenous young people from their frames of reference and experiences of reality, resulting in a phenomenological understanding of their perspectives and experiences of the early years of living with HIV. A total of 21 Indigenous young people took part open-ended interviews.

**Results:**

The stories that the Indigenous young people shared revealed their deeply interconnected social worlds, and how social determinants including abuse, trauma, being part of the child welfare system, and housing and food security were connected throughout various stages of their lives. Such stages included childhood, adolescence and young adulthood (the time of HIV infection), and later adulthood for older participants with the social determinants having multiple influences on their health trajectories.

**Conclusions:**

The findings highlight the need for policies and programs that are broadly focused, addressing multiple social determinants together. Overall, there needs to be more emphasis on the multiple social determinants in the life situations of all Indigenous youth. Reducing the health and social disparities in Indigenous youth is key to reducing the number of young Indigenous people diagnosed with HIV. The findings also shed light on the importance of listening to young Indigenous people who have experienced HIV diagnosis and life following diagnosis.

## Introduction

Disparities relating to race and ethnicity have been strongly linked to a variety of health inequities for youth [1]. Relating to the broader Canadian context, scholars suggest that an understanding of HIV-vulnerability in Indigenous peoples must begin with understanding the social determinants that are linked to the historical legacy of European colonization that has contributed to their suffering and unjust circumstances [2]. Indigenous youth are currently overrepresented in the HIV positive population [3]. HIV diagnosis during adolescence and young adulthood occurs at a time when individuals are forming their social identities and new relationships. It is also a time when young people are contemplating their futures. These factors warrant attention on the impact of HIV diagnosis on youth and their health and lives. However, minimal research exists on understanding Indigenous young people’s experiences of living with HIV during the critical period (i.e., the HIV diagnosis and early years following the HIV diagnosis). Indigenous youth also often must face an HIV diagnosis alone due to fear of stigma and discrimination, and are less likely to access services [4, 5]. It is clear that young Indigenous people living with HIV need to have their voices heard and be involved in identifying solutions to overcoming health and social disparities experienced by them. Furthermore, recent studies point to the need to understand the social determinants as they relate specifically to Indigenous youth [6, 7].

## Background

Persons of Indigenous descent (Métis, Inuit, and First Nations) are currently overrepresented in the HIV epidemic in Canada [3, 8]. While Indigenous people account for a mere 4.3% of the population [9], an estimated 6850 Indigenous people were living with HIV in 2014 (up 12.1% from 2011 estimates); that is, 9.1% of all HIV infections across Canada [8]. In the same year, an estimated 278 new HIV infections (10.8% of all infections) were reported among Canadian Indigenous peoples – a slight (1.7%) decrease from the figures estimated for 2011 estimate (250 to 450). This decrease may indicate a levelling-off in terms of overall representation of Indigenous peoples with regards to all new HIV infections. Indigenous people in Canada are also infected with HIV at a younger age than those who are not Indigenous [3]. Between 1998 to 2012, one-third (32.3%) of positive HIV test reports among the Indigenous were youth between 15 to 29 years of age, a percentage that well exceeds that of other ethnicities (20.6%). 72.2% of these youth identified as First Nation, 8.7% identified as Métis, 0.7% identified as Inuit, and 18.4% identified as “Indigenous unspecified.” Of the 950 reported cases of infection in Indigenous youth (15–29) in Canada between 1998 and 2012, 63% of the cases were due to intravenous drug use (IDU) [3]. 26.4% were due to heterosexual contact, of which 6.7% was men who have sex with men (MSM) and 3.6% MSM/IDU [3]. However, other social determinants can impact young Indigenous people. The broader social determinants that have been found to have health effects on Canadians include ‘Aboriginal status,’ disability, early life, education, employment and working conditions, food insecurity, health services, gender, housing, income and income distribution, race, social exclusion, social safety net, and unemployment and job history [10].

Negin et al. conducted a systematic review of the literature relating to HIV infection among Indigenous peoples in Canada, Australia, New Zealand, and the United States and found that domestic violence, stigma and discrimination, and injection drug use were the social determinants that were particularly important [7]. Many Indigenous youth including those with HIV are experiencing the trans-generational effects of colonialism, including the Residential School System, the ‘Sixties Scoop,’ and the on-going overrepresentation of Indigenous children in the child welfare system [11]. Residential Schools in Canada started in the 1870s and was a system of government funded, church-run schools that “were set up to eliminate parental involvement in the intellectual, cultural, and spiritual development of Aboriginal children.” The last Residential School closed in 1996 [12]. The ‘Sixties Scoop’ is a term coined by Patrick Johnson in the 1983 report *Native Children and the Child Welfare System*, which refers to the “mass removal of Aboriginal children from their families into the child welfare system,” which was particularly pronounced in the 1960s. Children were typically placed with middle-class Euro-Canadians [13].

The trans-generational effects of colonialism have contributed to the separation of families and the erosion of culture, in turn producing health and social disparities [14]. As is explained by Riediger (in McDonald), the Residential School System that removed Indigenous children from their families and tribal communities attacked “individual and cultural identity, and left alcoholism, suicide, violence, and ongoing sexual abuse in their wake” [15]. Kirmayer, Brass, and Valaskakis further note that the displacement or appropriation of land undermined Indigenous peoples’ spiritual connection to their environment, and has led to a fragmentation of identity and alienation from the world [16]. Finally, the historical trauma that has devastated many Indigenous communities appears to have resulted in a sort of “soul wounding” that is often transmitted across generations through transactions between parents and their children and social disadvantage [17, 18]. These realities in combination with several other aspects of the colonial encounter have eroded Indigenous “cultural buffers,” which have the potential to mediate youth’s vulnerability to negative health outcomes such as HIV infection [18].

Furthermore, given that adjusting to the diagnosis and learning to cope has serious health and social ramifications, research that details the experiences and needs of Indigenous people who were infected with HIV in their youth is critically needed. This article investigates the lived experiences of Indigenous people who became infected with HIV between the ages of 15 and 29. The research took place in Winnipeg, a mid-Western Canadian city with a small and steadily rising population of 750,000 people [19]. The city also has the highest proportion of Indigenous peoples for any Canadian city. Although limited in breadth and scope, the literature on the social determinants relating to HIV infection and health following diagnosis in young Indigenous people in Canada has centered on the Western (British Columbia) and Eastern (Ontario, Quebec) provinces, often to the neglect of Prairie Provinces such as Manitoba. HIV epidemiology on the Prairies and in Manitoba retains some important distinctive features. Hence, capturing the Manitoba perspective can add to and enrich existing research from other parts of Canada thereby contributing to a national model of young Indigenous people’s experiences of living with HIV/AIDS. Research situated in Manitoba is especially important because in 2015, 22.8% of new HIV infections were among Indigenous peoples [20].

## Methods

### Conceptual framework

Conceptual frameworks accounting for the intersectionality of social determinants have been deemed by scholars, health practitioners, and community people as appropriate ways for understanding the health trajectories and prevalence of HIV within racialized communities [21–23]. Hankivsky et al. describe intersectionality as “encouraging critical reflection that allows researchers and decision makers to move beyond the singular categories that are typically favoured in equity driven analyses (e.g., sex and gender in sex and gender based analysis) and also beyond the kind of enumerated list of determinants of health often found in health impact assessments to consider the complex relationships and interactions between social locations” (p. 2) [24]. Towards moving past singular categories, an anticategorical (denying fixed categories) or intracategorical (focusing on multiple social identities) approach to intersectionality can be used [25, 26]. We use the intracategorical approach in order to situate multiple social identities within the influence of the larger social structures [22, 25, 26], including related power dynamics, which must be accounted for in conceptually solid intersectional analyses [24, 27]. We frame power as operating within different structures that are affected by time and place [24], which is especially important for considerations around the lived experiences of Indigenous people who were infected with HIV at an early age and have had to navigate various systems affecting their wellbeing.

### Methodological approach

In order to ensure that the research is rooted in lived experience, an approach that supported the gathering of in-depth and detailed accounts of young people living with HIV/AIDS as opposed to standard survey methods was applied. The qualitative research design of phenomenology, as informed by van Manen, was used because it afforded the opportunity to understand Indigenous young people from their frames of reference and experiences of reality, resulting in a phenomenological understanding of their perspectives and experiences of the early years of living with HIV [28]. In using phenomenological research, the concern is with understanding or *Verstehen*, rather than causality or explanation [28]. To gain a full understanding of any phenomenon derived from an experience, the experience needs to be described as well as interpreted [28]. In order to ensure that the study was culturally-sensitive, this study was also guided by a participatory research approach which supported the full and active participation of the community being researched, and involved seeking and respecting the knowledge and expertise of community members (i.e., collaborators, Indigenous youth living with HIV and their support persons, and health and social care service providers) and support personnel throughout the entire research process including research design, data collection and analysis, and knowledge translation [28, 29].

### Recruitment and participants

A number of strategies were used to recruit the participants. First, invitation letters were distributed to potential participants via a designated intermediary by three of the study’s collaborators: Nine Circles Community Health Centre, a community based, non-profit community health and community lead site for the Manitoba HIV Program; Ka Ni Kanichihk Inc., a registered non-profit corporation whose mandate is to provide Indigenous identified programs and services that focus on wholeness; and the Health Sciences Centre (Medicine Program), the Tertiary lead site for the Manitoba HIV. Second, the study was advertised through the use of posters, newsletters, and websites at all of the collaborators’ sites as well as other community-based health and social service agencies that provide services for Indigenous people living with HIV. We used both purposeful and snowball sampling techniques [30, 31]. Interviews were conducted until data saturation was reached [31]. A total of 21 participants took part in the study, 10 male and 11 female. The median age of HIV infection was 24 years of age. The demographic form also asked participants to rank their health status and overall quality of life. Demographic and health status information are reported in Table 1.

**Table 1:** Demographics, and health and well-being information for Indigenous people who became HIV positive between the ages of 15 and 29

Participants: Indigenous people who contracted HIV between 15 and 29					
Gender	*N*	*%*	Health in past year	*N*	*%*
Female	11	52.4	Excellent	2	9.5
Male	10	47.6	Very good	5	23.8
Age			Good	1	4.8
20–25	3	14.3	Fair	7	33.3
26–30	6	28.8	Poor	4	19.1
31–35	5	23.8	Very poor	0	0
36–40	1	4.8	Other / unsure	2	9.5
41–45	6	28.6	Health in past month		
Age when diagnosed with HIV			Excellent	3	14.3
15–18	2	9.5	Very good	5	23.8
19–21	1	4.8	Good	1	4.8
22–25	7	33.3	Fair	4	19.1
26–29	8	38.1	Poor	6	28.6
Other / uncertain	3	14.3	Very poor	0	0
Sexual orientation			Other / uncertain	2	9.5
Lesbian	0	0	Quality of life in past year		
Gay	2	9.5	Excellent	2	9.5
Heterosexual	15	76.2	Very good	1	4.8
Bi-sexual	0	0	Good	6	28.6
Two-spirited	2	9.5	Fair	4	19.1
Other	2	9.5	Poor	5	23.8
Education			Very poor	2	9.5
Grade 9 and under	2	9.5	Other / unsure	1	4.8
Grade 10 - some 12	7	33.3	Quality of life in past month		
High school	4	19.1	Excellent	3	14.3
Post-secondary	6	28.6	Very good	3	14.3
Other / no response	2	9.5	Good	6	28.6
Employment			Fair	4	19.1
Yes (part-time)	1	4.8	Poor	3	14.3
No	17	81.0	Very poor	1	4.8
Volunteer	2	9.5	Other / uncertain	1	4.8
Other / uncertain	1	4.8			

### Data collection

Interviews with Indigenous people who had become infected with HIV during their youth were conducted in two sessions. When considering social and health related issues, “youth” is considered to be the period of life between 15 and 29 years of age [32, 33]. This is consistent with the norms in social and health related research [32], and the age range used by the Public Health Agency of Canada [3, 8]. Based on the recommendations of our community partners, a young Indigenous woman, with a background in social work and experience in working with vulnerable populations, was recruited to conduct the interviews. Prior to the commencement of data collection, the interviewer underwent a comprehensive training session led by the first author, an expert in qualitative research methods.

Participants took part in two interview sessions so that they would have adequate time to reflect and tell their stories. In order for us to arrive at a deeper understanding of the phenomenon under study, use of the open-ended interview technique was used for interview session. The open-ended questions and interview technique gave study participants the opportunity to discuss what they considered important, to have greater control in the interview process and to narrate their experiences without being tied down to specific answers [34]. For the first interviews, the opening questions were, “Can you please tell me a little bit about yourself?” followed by, “Can you please tell me what life was like before and after being diagnosed with HIV?” From there, initial answers are probed until the experience was comprehensively described. They were then asked to talk about how their life might have changed during three periods: the period immediately after being diagnosed with HIV; the period during the first year of living with HIV; and the subsequent years after the first year of living with HIV. Each interview lasted from 60 to 90 min. All interviews were digitally recorded and transcribed verbatim to preserve their authenticity. After each interview, field notes were recorded and transcribed describing nonverbal behaviours, communication processes, rapport, interview context, and procedural problems. These field notes also served as a space for reflection for the interviewer, to check for and acknowledge any personal biases and offered an opportunity for the interviewer and first author to debrief.

### Data analysis

We used van Manen’s method of data analysis to analyze the data generated from the study participants [28]. van Manen’s method of data analysis involves delineating units of meaning from the data, clustering units of meaning to form thematic statements, and extracting themes [28]. Interview data and field notes were first reviewed repeatedly by the first three authors for significant statements in an attempt to find meaning through themes [28]. Thematic statements were then isolated by selecting and highlighting sentences or sentence clusters that stand out as thematic of the lived experiences of study participants. Using all the phrases and sentence clusters, textual data was then reduced until themes emerged that provided understanding of the social determinants of HIV infection and health following diagnosis for the study participants. Preliminary themes were distributed to the research team and community partners, each with diverse backgrounds. After discussion with the research team, findings from the participants were then compared to findings from the literature that addressed the social determinants impacting Indigenous young people (e.g. intravenous drug use, partner abuse, etc.).

### Ethics

Before commencing the study, permission was obtained from the Assembly of Manitoba Chiefs Health Information Research Governance Committee, University of Manitoba Research Ethics Committee, and from the recruitment sites. Consent from all participants was also obtained. Participants were informed that they could withdraw from the study at any stage. Strategies to secure the participant confidentiality were applied. All participants received an honorarium gift card for their participation and mental health supports were put in place for participants who may have wished to access them.

## Results

**Fig. 1:**
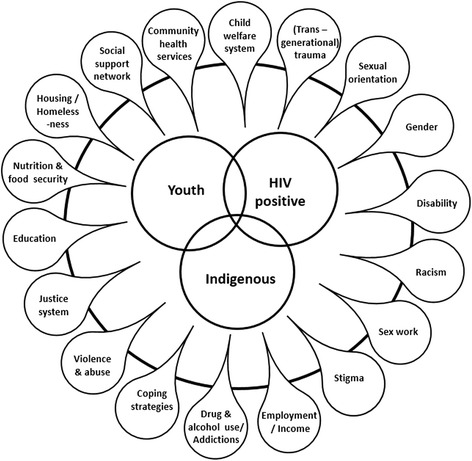
The multiple intersecting social determinants of health for Indigenous people who were infected with HIV as youth in Manitoba, Canada

Through thick description and interpretation, the essential meaning of what it is like to be an Indigenous person infected with HIV in their youth resulted. Several themes were identified by study participants as being important social determinants of health both prior to and following HIV infection (Fig. 1). These social determinants are detailed in Table 2, and stories from four study participants (pseudonyms are used) are shared towards illustrating the intersectional quality of the social determinants.

**Table 2:** The multiple intersecting social determinants of health for Indigenous people who were infected with HIV as youth in Manitoba, Canada

Social determinant	Experiences prior to infection (as youth)	Experiences after infection (from youth to later years)
*Child welfare system*	• Growing up in care	• Having children taken into care
*(Trans-generational) trauma*	• Parents not knowing how to parent (reflections on the Residential School System as eroding parenting)	• Reflections on trauma from different stages of development • Trauma from experiences later in life (e.g., late-term miscarriages)
*Sexual orientation*	• LGBTQ2* youth not being able to be “out” (express sexuality freely) in the community (urban and on-reserve)	• HIV positive LGBTQ2* youth and adults not always (only sometimes) being able to find the appropriate spaces for care (e.g., group counseling) • Lack of resources for HIV positive transgendered Indigenous people, including youth
*Gender*	• Gender violence • Sexual assault	• Gender violence • Access to gender specific HIV-support programming
*Disability*	• Learning disabilities (e.g., ADHD)	• Learning disabilities (e.g., ADHD) • HIV-related disability
*Racism*	• Experiences with racism when accessing health services (e.g., lack of understanding of Indigenous culture)	• Experiences with people (i.e., family, friends, and other people in the community) thinking HIV is an “Aboriginal disease”
*Sex work*	• Selling sex as a survival mechanism • Being trafficked by pimps as a child	• Continued involvement in the sex trade • Discontinuation of involvement in the sex trade
*Stigma*	• Not discussing HIV because it is taboo and considered to be something only “dirty” people have	• Not being able to access services in First Nations communities because of heavy stigma around HIV and fear that personal information will be spread around the community
*Employment / Income*	• Full-time work • Part-time work • Unemployment • Low-to-medium income	• Unemployment • Part-time work • Volunteering • Low income
*Drug and alcohol use / Addictions*	• Drug and alcohol use • Addiction • Sobriety	• Drug and alcohol use • Addiction • Sobriety
*Coping strategies*	• Drug and alcohol use • Friends and family	• Drug and alcohol use • Friends and family • Music and art • Journaling • Cultural practice (e.g., ceremony) • Humour
*Violence / Abuse*	• Physical and/or sexual abuse from parents and step-parents • Abusive partner • Gender violence and sexual assault	• Abusive partner • Violence from strangers (especially for those who are homeless)
*Justice system*	• Arrest • Incarceration (juvenile detention)	• Arrest • Incarceration
*Education*	• In high school • Finished high school • Dropped out of high school • Some health and sexual education through school	• Finishing high school • Training programs • Post-secondary education • HIV specific education
*Nutrition and food security*	• Low food security because of instable home environment or homelessness • Medium to high food security through stable home environment	• Low food security due to poverty and homelessness • Medium to high food security due to own income • Improved food security due to access to food banks • Attention to special HIV supportive diet
*Housing / Homelessness*	• Residing with parents • Independent living • Periodic and prolonged homelessness • Residing with foster parents	• Stable in depending housing • Shared housing (e.g., rooming houses) • Periodic and prolonged homelessness • Incarceration
*Social support network*	• Family and friends • Teachers	• Family (including children) and friends • Partner • Health providers (Indigenous and non-Indigenous) • Church and religious community
*Community health services*	• For participants living on reserve, limited access and travel required to access health services, especially for specialized services • Fear of accessing local services due to stigma in the community and fear that information will be shared • Fear of accessing services due to their location (not wanting to see certain people, have street drugs offered to them, encounter violence, etc.) • Lack of information/education on HIV prevention prior to diagnosis	• Both positive and negative experiences with nurses and doctors at hospitals and clinics • HIV management programs • HIV medications • Mental health counseling • Culturally appropriate services (e.g., through Tribal Nursing Officer)

*LGBTQ2 stands for lesbian, gay, bi-sexual, transgendered, queer and two-spirited. Two-spirited is an Indigenous concept for understanding people who possess both the male and female spirit, and do not identify discretely with either one gender

### Anita’s story (AY17)

Anita’s (35 years old at the time of the interview) life has been punctuated with many challenges to her health and well-being since she was a child, which can be connected to her eventual HIV diagnosis at the age of 18. Anita was sexually abused by her stepfather as a young child. Before the age of 10, in order to escape from her family life, Anita started sniffing solvents and hanging out with older girls. Those girls taught her how to make money by “going into cars” with men. Before reaching her teenage years, Anita started dating a man in his twenties. It was not long before this boyfriend started trafficking her. Over the years, she experienced multiple rapes and violence from clients. Eventually, Anita started using multiple substances including intravenous drugs to cope with being sold for sex as a child and being physically abused by her pimp who also provided her with substances to feed her addictions. Anita’s life took another turn when her pimp took her aside and taught her to fight back with a weapon (a knife). Anita described the experience of physically defending herself from clients as “feeling like I was in control.” From this point, Anita continued down the path towards other forms of violence, developed a reputation as a fighter, and a position with dealers as a debt collector. She was charged for multiple violent robberies from her clients and was sentenced to 5 years in prison, which she believes was a reduced sentence because of her personal history and belief that she was doing the right thing by punishing men who were buying sex from children and young women.

Anita found out that she was HIV positive when she was in prison. She went to get tested for Hepatitis C, which she feared she had contracted through sharing needles with a friend. This is when she found out that she had both Hepatitis C and HIV.

“We were both in jail and we were walking around and having our yard time and that’s when she told me, she’s like “You know, you should get tested.” She started crying and that’s when she told me she had Hepatitis C and that I should get tested, so I just figured okay well I’ve got Hepatitis C to worry about, and all of a sudden I got the whole works done and then I find out that I have HIV.”

At the time of the interview Anita was no longer in prison and was in a committed relationship with a man who is also HIV positive. She has two children (who are not HIV positive), both of which were taken into care at birth.

### Danny’s story (AY04)

Danny’s relationship with his father was tumultuous as a child. His father was an alcoholic, and abused him physically and mentally. In his 20s, Danny started standing up for himself through enacting similar violence on his father. He would be asked to leave the home following the conflicts, which led to periods of homelessness.

“Pulled my hair one times in front of my friends. I did that to him and beat him up. ‘Get up man’ you know, that’s what he used to say to me. He was like ‘Stand up to me, be a man, be a man,’ and here I am just a kid like that.”

Just prior to becoming infected with HIV at 24 years of age Danny (26 years old at the time of the interview) described his lifestyle as healthy. He was going to the gym almost every day and stopped using street drugs (marijuana). Danny was very sexually active during this time and had multiple partners. Following diagnosis, Danny used journaling and music to cope with his emotions. Danny found many of his old relationships with friends changed following his diagnosis, and that many people simply did not understand HIV. Danny became more and more disenfranchised from his friends in the year following his diagnosis and eventually started selling drugs for one of his remaining friends. He talked about homelessness and food insecurity during this (recent) period of his life. After trying to navigate on his own for several months, Danny contacted a youth support agency that guided him to get registered for social assistance. He expressed that he felt like the people at the agency treated him with respect at first, but then he felt they became less tolerant and conflicts with the social worker led him to stop accessing services. At the time of his interview, Danny was accessing HIV-specific services from a different agency, a food bank, and was seeing a nutritionist.

### Jolene’s story (AY03)

Jolene grew up on-reserve, and described being estranged from her parents for some time (they did not know about her HIV positive status or the birth of her baby). At the time of the interview, Jolene was living in Winnipeg and said that she did not wish to return to living on reserve because she did not want to be close to certain issues, such as suicide. Jolene was 23 when she was diagnosed with HIV (25 at the time of the interview). She believes that she contracted HIV through a violent incident with a knife where someone was cut prior to her being stabbed. Jolene was not tested for HIV directly following this violent incident, and found out about her positive status during routine blood tests during her pregnancy. Jolene describes “feeling nothing” at the time when she was diagnosed and that she still doesn’t fully understand HIV or what it does. Jolene stated that there was a lack of information following her diagnosis, and that she was afraid to ask or do her own research.

“I don’t really know HIV… (chuckle) What does it do?”

**Interviewer:** “And you never did any research yourself like to go look it up like Google.”

“No. If I, if I know those, if I know what is, what is it...I get scared (chuckle)…That’s why I don’t want to ask” (chuckle).

Jolene had not attended any HIV programs, and did not know of any at the time of the interview. Jolene’s baby was taken into the child welfare system at birth. She believed this happened because the child welfare system does not understand parents with HIV, and that she had done everything right to prepare for being a parent. Jolene’s baby took medication directly following her birth in order to prevent becoming infected with HIV. The baby was tested for HIV after several months and the test results were negative. Jolene had only disclosed her positive status to her best friend and her partner at the time of the interview because she felt that they were the only people that she could trust.

### Sky’s story (AY23)

Sky’s parents were not present in childhood and Sky lived with his grandmother on-reserve until 16 years of age when he moved to Winnipeg to live with cousins. When he moved to the city, Sky felt the freedom to express a different side of his gender (Sky identified as male at the time of the interview). Sky, who had been expressing as male up until this point, started to express female beginning by dressing in female clothes. Sky also started going to LGBTQ2 (stands for lesbian, gay, bisexual, transgendered, queer and two-spirited) friendly nightclubs. At 16 years old, to make money, Sky started participating in the sex trade. Two years later, at 18 years old, Sky left the sex trade and decided to live as a male. Sky met the person who would become his long-term partner. Sky received an HIV-positive diagnosis at 26 years old (30 at the time of the interview) through an Indigenous health centre. Sky believe that he contracted HIV from having sex with another person outside of his relationship whose status became known to him at a later date. Sky tried to break up with his partner following his diagnosis because he felt guilty about being unfaithful to the relationship and contracting HIV, but his partner, who is not HIV positive, wanted to remain in the relationship. Sky said that he feels supported by his partner, as well as his family who he believes learned about HIV through their experiences with a transgendered family member who passed away from HIV related complications.

“I have a cousin that passed away from HIV. And um they didn’t treat him really nice.”

**Interviewer:** “The family?”

“Yeah, and you know what I think now, I think that like she like paved a way for me somehow, like or ‘cause she lived transgender, she was a transgendered woman. So, she was living like that every day and she would come home and everybody liked her. She was funny and everybody liked her around, but it was weird how they were. How they were treating her like, like washrooms, that was a big deal. She wouldn’t even be able to use the washroom there.”

Since diagnosis, Sky has felt physically very healthy, which he attributes to having access to the right medication (receives government assisted support because of high cost) and health services (e.g., good relationships with service providers), being sober and his active lifestyle (dancing, yoga and other activities). Sky uses humour and traveling to visit family to cope, and wants to start a group for HIV positive men.

## Discussion

Our study highlights the intersectional social determinants of HIV infection and life beyond diagnosis for young Indigenous people in Manitoba, Canada. The stories that the participants shared revealed their deeply interconnected social worlds, and how social determinants were connected throughout various stages of their lives. Such stages included childhood, adolescence and young adulthood (the time of HIV infection), and later adulthood for older participants. The participants who shared their stories and lived experiences with us and were often highly capable of understanding the behaviours that put their health at risk [35], as well as the intersectional nature of the social determinants of health [36]. Through an intracategorial approach to understanding intersectionality and listening to the stories of young people who had become infected with HIV in their youth, it was possible to account for the multiple interconnected social determinants that were associated with HIV infection and overall health trajectory prior to and following HIV diagnosis [22, 23, 26].

Social determinants like intimate partner violence and other types of abuse were associated with the participant’s stories about the years leading up to and early years following diagnosis. These interrelated factors have been linked to the burden of HIV infection among Indigenous youth in areas across Canada, and in particular have affected young Indigenous women [37–40]. Studies outside of Canada have also revealed a strong connection between HIV infection in young women and partner violence [41–43]. It is estimated that nearly half (47.3%) of all positive HIV test reports of Indigenous people in Canada were female between 1998 and 2012, compared to the 31.6% among their non-Indigenous counterparts [3]. In 2015, Manitoba continued to have the highest proportion of new cases of Indigenous female HIV infection in Canada [20]. Young Indigenous women appear to be more susceptible to HIV infection than their male counterparts due to sex and drug-related harm. Parallel to our findings, Mill found that female Indigenous youth from across Canada tended to associate the sexual abuse they often endured in early childhood with risky sexual behavior such as early sexual contact, unsafe sex, and prostitution [37]. Maté, former physician of Vancouver’s beleaguered Downtown Eastside, also observed that the effects of sexual abuse often led Indigenous women to serious forms (intravenous) of drug use in their struggle to overcome suffering [44]. Work by Stevens et al. revealed that an HIV diagnosis was experienced as a ‘traumatic event’ in women that caused a rift in their sense of identity and resulted in them facing unrelenting misery, escalated drug and alcohol use, depression, suicidal behaviours, transmission risks, destabilization of relationships, and lowered income and shelter during the early years [45].

In one study among low-income women in Baltimore, there was a “cumulative and syndromatic relationship among commonly co-occurring vulnerabilities (homelessness, incarceration, low-income, residential transition) in association with HIV-related risk behaviors” [46]. This was the case for the participants, both male and female, who were engaged through this study. Access to food and housing was especially important for the participants, some even making direct connections between their ability to manage their illness with medications (ART) and their ability to have food (especially healthy food) and housing (especially that which was affordable and sustainable). Work by other scholars has also determined the impact of affordable and sustainable housing on the health outcomes [47, 48], including the material, meaningful and spatial dimensions of housing on both physical and mental health [49]. Furthermore, German and Latkin found that “social policies and programs aiming to enhance housing and overall social stability are likely to be beneficial for HIV prevention” [46] (p. 168). Study participants also connected housing with food (i.e. having a place to cook) and talked about their overall need to access foods, which also connected them with health (HIV) programs and services that were offered in the same places as food banks. A community food security assessment by Zurba et al. in Manitoba’s main urban centre (Winnipeg) found that access to healthy and culturally appropriate foods was connected to reducing drug use and other social determinants of health, and was important for overall emotional well-being [50]. Education was also an important factor in HIV prevention and well-being following diagnosis. Reducing the stigma around HIV and providing thorough information in culturally appropriate formats will be important for affecting the health trajectories of Indigenous youth following diagnosis.

Many participants were aware of the effects of colonial systems on their health trajectories. The strong connections existing between social determinants of HIV and health following diagnosis run parallel with those associated with the Residential School System, the ‘Sixties Scoop,’ and the on-going overrepresentation of Indigenous children in the child welfare system [51]. Negin et al. emphasised the effects of colonization and marginalization on HIV-related behaviors, especially domestic violence, injection drug use, and stigma and discrimination [7]. Several Indigenous scholars agree that the trans-generational trauma that is created by the breaking apart of families will need to be addressed through various forms of action within the health, child welfare and other institutions if Indigenous peoples in Canada (and other places with similar colonial histories) are to have improved health outcomes [52–56]. Many also argue that institutional transformation cannot only come through simple changes in policy [57], but will require pathways within institutions to make way for Indigenous resurgence through nation-building, reconnection to homelands, integration of culture and spirituality, leadership, and decolonized forms of decision-making [56, 58–60]. Similarly, a qualitative study by Larkin et al. found that young Indigenous people (from Ontario) were more aware of structural inequities that contribute to HIV risk than those who were not Indigenous [2]. The participants of this study also understood the interconnections between social determinants, which were often multiple, with some determinants being deeply entangled and more pronounced during different stages of life, from birth through early adulthood.

### Strengths and limitations

A strength of this research is that it explores the social determinants of HIV infection and health following diagnosis from the perspectives of Indigenous people who had become infected with HIV during their youth. While we strived to also recruit younger participants (15–19 years of age), a limitation of this study was that the participants were all over the age of 20 at the time of the research. Our inability to recruit younger participants may be related to it being a very vulnerable time in their lives; fear and self-stigma may have contributed to teens not getting involved in the study. Conducting research with HIV positive Indigenous teens might produce different results because they would be reflecting on more recent circumstances, instead of those from several years ago. Also warranted is longitudinal work in order to capture how the perspectives of young people change over time as influenced by the changing social determinants.

## Conclusions

Our study considered the intersecting social determinants of HIV infection and health following diagnosis from the perspectives of Indigenous people who had become infected with HIV as youth. Study participants were often able to identify the social determinants and how they were interconnected throughout different stages in their lives. The findings highlight the need for policies and programs that are broadly focused, addressing multiple social determinants together. Overall, there needs to be more emphasis on the multiple social determinants in the life situations of all Indigenous youth. Reducing the health and social disparities in Indigenous youth is key to reducing the number of young Indigenous people diagnosed with HIV. The findings also shed light on the importance of listening to young Indigenous people who have experienced HIV diagnosis and life following diagnosis. By situating young Indigenous people’s perspectives at the centre of investigation, a deeper understanding of how to best meet their needs may be advanced and will contribute to the development of meaningful and culturally-sensitive services and programs for young Indigenous people living with HIV.

## Abbreviations

AIDS: Acquired immunodeficiency syndrome
HIV: Human immunodeficiency virus
LGBTQ2: Lesbian, gay, bi-sexual, transgendered, queer and two-spirited
